# Physician, Nurse, and Advanced Practice Provider Perspectives on the Rapid Transition to Inpatient and Outpatient Telemedicine

**DOI:** 10.1089/tmr.2021.0034

**Published:** 2022-01-10

**Authors:** Katherine A. Meese, Allyson G. Hall, Sue S. Feldman, Alejandra Colón-López, David A. Rogers, Jasvinder A. Singh

**Affiliations:** ^1^ Department of Health Services Administration, The University of Alabama at Birmingham, Birmingham, Alabama, USA.; ^2^ UAB Medicine Office of Wellness, Birmingham, Alabama, USA.; ^3^ Healthcare Quality and Safety Programs, Department of Health Services Administration, The University of Alabama at Birmingham, Birmingham, Alabama, USA.; ^4^ Center for Outcomes and Effectiveness Research and Education, The University of Alabama at Birmingham, Birmingham, Alabama, USA.; ^5^ Department of Health Services Administration, School of Health Professions, Heersink School of Medicine, The University of Alabama at Birmingham, Birmingham, Alabama, USA.; ^6^ Department of Sociology, The University of Alabama at Birmingham, Birmingham, Alabama, USA.; ^7^ UAB Medicine Chief Wellness Officer, Birmingham, Alabama, USA.; ^8^ Department of Surgery, ProAssurance Chair of Physician Wellness, Heersink School of Medicine, The University of Alabama at Birmingham, Birmingham, Alabama, USA.; ^9^ Department of Medicine, Heersink School of Medicine, The University of Alabama at Birmingham, Birmingham, Alabama, USA.; ^10^ Department of Epidemiology, The UAB School of Public Health, Birmingham, Alabama, USA.; ^11^ Medicine Service, VA Medical Center, Birmingham, Alabama, USA.

**Keywords:** telemedicine, COVID-19, physician, nurse, advanced practice provider, inpatient, outpatient, team

## Abstract

**Background:** Many health systems transitioned rapidly to using inpatient and outpatient telemedicine during the COVID-19 pandemic. Prior research has examined clinician satisfaction and experiences with telemedicine in a siloed approach for specific provider types. Less is known about how experiences with the rapid transition to telemedicine affected the entire clinical team, and how this contributed to their overall distress.

**Methods:** A survey was conducted within a large academic medical center in the Southeastern United States during June of 2020. The survey asked about experiences with inpatient and outpatient telemedicine and overall distress. Analysis of variance was calculated to examine differences in experiences among physicians, nurses, and advanced practice providers (APPs) with both inpatient and outpatient telemedicine. Multivariate regression analysis was conducted to determine whether reported telemedicine stressors were associated with changes in overall distress scores. Qualitative comments provided during the survey were included to illustrate the quantitative findings.

**Results:** Of the 1130 survey respondents, 237 indicated that they used telemedicine. Telemedicine use was not statistically significantly associated with overall distress scores. The APPs indicated the greatest satisfaction with telemedicine, followed by physicians and then nurses. Team members differed on their perceptions of quality of care and safety for inpatient and outpatient telemedicine. Physicians (70%) and APPs (64%) felt safer having the option to use inpatient telemedicine, whereas only 26% of nurses reported the same. Overall, >70% of physicians and APPs would like to continue having the option to use inpatient and outpatient telemedicine in the future, whereas <50% of nurses reported the same.

**Discussion:** These results suggest that telemedicine holds promise for providing care beyond the pandemic, and it may be a mechanism to improve flexibility, autonomy, and expand patient access. Implementation of new technologies must consider the experiences of the entire team, rather than a siloed approach to determining satisfaction with the changes.

## Introduction

Inpatient and outpatient telemedicine use surged in 2020 during the early months of the COVID-19 pandemic. Although telemedicine became an increasingly popular mechanism for outpatient care during the lockdown across U.S. states and cities, many medical centers also adopted inpatient telemedicine.^1–4^ The Centers for Medicare and Medicaid Services, state Medicaid programs, and private insurers made regulatory and payment changes to encourage increased use of telemedicine services.

The current literature suggests that clinicians and patients and their families are generally satisfied with inpatient telemedicine. For example, inpatient telemedicine services were effective in establishing an accurate dermatology diagnosis and in facilitating the coordination of psychiatric care.^3,5^ Also, high levels of satisfaction and perceptions of quality of care among both parents of pediatric patients and rural health care providers were associated with inpatient telemedicine use.^6^

Studies examining the role of telemedicine suggest a reduction in patient transfers from hospital facilities (e.g., remote, face-to-face), length of stay, reduction in the median duration of antibiotic treatment, and similarities in transfer to the Intensive Care Unit (ICU), number of ICU days, and ventilation between patients with and without telehealth visits.^2,7^ Providers who support virtual outpatient visits see telemedicine as a convent tool, because it contributes to public health safety and the cost of care and transportation.^8–13^

Provider concerns associated with telemedicine include the inability to complete a physical examination, to order interventional procedures, and to manage complex medical problems. Providers have also reported concerns related to reimbursement and medicolegal liability.^14^

During the COVID-19 pandemic, one of the objectives of telemedicine was to minimize the risk of exposure among frontline health care workers in outpatient and inpatients units and aid the conservation of personal protective equipment (PPE).^4,15^ Telemedicine serves as a safe health care delivery alternative that allows patient–provider interactions without putting both parties at risk.

The pandemic appears to be settling into longer-term endemic situation, with spikes and declines of infection rates.^16^ Although outpatient telemedicine use plateaued over time, the number of virtual visits has not returned to pre-pandemic levels and upticks appear to be simultaneous with more infections.^17^ Therefore, there is a reasonable expectation that the adoption and use of telemedicine will continue and likely remain a key tool in health care.

For some organizations, transitioning to the telemedicine format did not happen smoothly due to low or no training, inadequate access to electronic equipment (e.g., computer, camera), and lack of technical support.^2,18^ In addition, telemedicine has been criticized because it limits the ability of clinicians to use all of their senses to understand patients' complaints and develop trusting relationships with them. This can be associated with increased stress and negative job satisfaction among frontline providers.^19^

As the adoption of telemedicine becomes more mainstream, an important consideration are the experiences of frontline health care workers and the degree to which the modality contributes to work-related stress or work-related well-being. In addition, the experiences and level of stress or sources of frustration may differ between nurses, advanced practice providers (APPs), and physicians.^20^

Therefore, in this article, we examine the experience, satisfaction, and frustrations associated with both inpatient and outpatient telemedicine from the perspective of various health care workers, including physicians, nurses, and APPs, at a large academic medical center. In addition, we examine the relationship between perceptions of clinician well-being and the number of frustrations and perceived positive aspects of telemedicine.

## Methods

### Study design

Data were collected as part of a larger anonymous cross-sectional survey study of employees within a large academic medical center in the Southeast United States, and they were approved by the organization's Institutional Review Board. In June 2020, an optional online employee survey was sent to 6276 medical center employees. For 3 weeks, we sent invitation emails encouraging potential participants to complete the survey, which opened in the middle of June and closed in the middle of July.

Participation was voluntary and anonymous. A total of 1130 respondents took the survey, including 588 physicians, APPs, and nurses, with a response rate of 18%. Cases missing key variables of interest were excluded from the analysis (*n* = 346). Of those respondents, 237 people reported using either inpatient or outpatient telemedicine or both.

*The survey measured individual factors such as distress and work-related stressors and organizational-level factors, as well as experiences with both inpatient and outpatient telemedicine. They were asked about their overall satisfaction with telemedicine, and their perceptions of the quality of care and patient experiences with using telemedicine. The questions were based on feedback from physicians and nurses using telemedicine collected during unit rounding, as well as the executive leadership for telemedicine initiatives. The participants were also asked about what specifically was going well with telemedicine, their specific frustrations with telemedicine, and what overall positive changes had occurred at work during COVID-19.

Multivariate regression analysis using listwise deletion was conducted to determine whether telemedicine use or frustrations with telemedicine were associated with overall distress using the validated Well-Being Index score.^21–24^ Analysis of variances were conducted to determine whether APPs, nurses, and physicians differed in their experiences using inpatient and outpatient telemedicine. In addition, qualitative open-ended responses asking for feedback on telemedicine were collected and categorized into different themes using axial coding strategies by the primary and secondary authors.^25^

## Results

### Telemedicine and distress

Our sample was mainly composed of health care workers between 40 and 50 years old (Table 1). The majority of participants within each job role and for the entire sample identified as female, White, and non-Hispanic. When asked to identify specific clinical stressors, 51% of APPs using telemedicine indicated that telemedicine was a major clinical stressor, whereas only 26% of nurses and 38% of physicians noted the same (Table 2). Despite indicating telemedicine as a major clinical stressor, in the multivariate regression analysis, inpatient telemedicine, outpatient telemedicine, or using both was not statistically significantly associated with overall distress scores (Table 3).

**Table 1.** Sample Characteristics (*N* = 201)

	APP (*N* = 106), %	Nurse (*N* = 34), %	Physician (*N* = 61), %	Total, %)
Age, M (SD)	41.74 (12.53)	42.29 (13.12)	48.43 (15.18)	44.12 (13.85)
Gender				
Male	9.43	11.76	44.26	20.40
Female	79.25	79.41	47.54	69.65
Self-describe	0.00	2.94	0.00	0.50
Prefer not to answer	11.32	5.88	8.20	9.45
Race				
Non-Hispanic White	73.58	76.47	78.69	75.62
Non-Hispanic Black or African American	7.55	8.82	3.28	6.47
Hispanic or Latinx	0.00	0.00	4.92	1.49
Native American or Alaskan Native	0.00	2.94	0.00	0.50
Asian	0.94	0.00	4.92	1.99
Two or more races	0.94	0.00	0.00	0.5
Self-identify	1.89	0.00	0.00	1.00
Prefer not to answer	15.09	11.76	8.20	12.44

%, percent within the job role group and total sample; APP, advanced practice provider; M, mean; SD, standard deviation.

**Table 2.** Descriptive Statics of Well-Being Index Score Predictors (*N* = 201)

	APP	Nurse	Physician	Total	*p* ^a^
% Telemedicine as a clinical stressor^b^	50.94	26.47	37.70	42.79	7.22^*^
Average count of positive changes brought by telemedicine^c^	3.85	3.12	3.44	3.60	1.81
Average count of telemedicine frustrations^c^	3.87	4.47	4.85	4.27	2.85

^*^ *p* < 0.05.

^a^ Significance of ANOVA or Chi-squared tests determining the association between telemedicine as a clinical stressor, average count of positive changes brought by telemedicine, or telemedicine satisfaction.

^b^ Chi-squared test.

^c^ ANOVA.

ANOVA, analysis of variance.

**Table 3.** Ordinal Least-Square Regression Predicting Well-Being Index Score (*N* = 201)

	β (95% CI)
Telemedicine as a clinical stressor	0.101 (−0.563 to 0.766)
Total count of telemedicine frustrations	0.123 (−0.001 to 0.247)
Total count of telemedicine positives	−0.021 (−0.165 to 0.122)
Telemedicine use (Ref. = Outpatient)	−0.077 (−1.161 to 1.008)
Both	
Inpatient	−0.030 (−1.070 to 1.010)
Work location (Ref. = Administration or Other)	
Ambulatory	0.118 (−0.837 to 1.073)
ICU	1.703 (0.043 to 3.350)^*^
Non-ICU	−0.056 (−1.216 to 1.104)
Operating room or procedural	0.267 (−1.044 to 1.578)
Job category (Ref. = Physician)	
APP	1.240 (0.542 to 1.939)^**^
Nurse	2.012 (1.185 to 3.058)^***^

^*^ *p* < 0.05, ^**^p < 0.01, ^***^p < 0.001.

CI, confidence interval.

Of the 237 people who used telemedicine, 55 (23%) specifically mentioned telemedicine as a positive intervention or change that had occurred during COVID-19, as represented by the following quote:

Telemedicine has been wonderful for providers as well as patients. My stress was greatly reduced once our clinic closed [because] I could work from home and monitor my children to be sure they were at home and doing their online school. [APP]

### Clinician satisfaction

The various members of the care team differed on their overall satisfaction using telemedicine, for both inpatient (*F* = 6.06, *p* < 0.05) and outpatient (*F* = 5.89, *p* < 0.05). Overall, APPs had the highest inpatient telemedicine satisfaction scores (3.61 out of 5), with nurses (2.72 out of 5) and physicians (2.73 out of 5) ranking the lowest (Table 4). Sixty percent (*n* = 14) of APPs agreed or strongly agreed that they were satisfied with their experience using inpatient telemedicine, whereas <30% of physicians (*n* = 4) and nurses (*n* = 6) said the same.

**Table 4.** Likert Score, Percentage, and Analysis-of-Variance Test Describing Advanced Practice Providers’, Nurses’, and Physicians' Inpatient and Outpatient Telemedicine Use

**Inpatient telemedicine**							
	**APP**		**Nurse**		**Physician**		***F***
Number of observations	29		21		17		
	**Score^a^**	**%^b^**	**Score^a^**	**%^b^**	**Score^a^**	**%^b^**	
I am satisfied with my experience using inpatient telemedicine	3.61	61	2.72	22	2.73	27	6.06^*^
I would like to continue to have the option to use inpatient telemedicine in the future	3.96	79	3.29	47	3.87	67	2.73
I felt safer having the option of using telemedicine to treat patients during COVID-19	3.92	75	2.88	29	4.07	64	11.35^*^
I am comfortable with the quality of care patients are receiving when I am using inpatient telemedicine	3.54	63	2.65	35	2.93	29	3.55^*^
**Outpatient telemedicine**							
	**APP**		**Nurse**		**Physician**		***F***
Number of observations	99		33		60		
	**Score^a^**	**%^b^**	**Score^a^**	**%^b^**	**Score^a^**	**%^b^**	
I am satisfied with my experience using outpatient telemedicine	3.80	70	3.04	31	3.53	60	5.89^*^
I am comfortable with the quality of care NEW patients are receiving when I am using telemedicine	3.37	48	2.89	30	3.11	41	2.15
I am comfortable with the quality of care existing patients are receiving when I am using telemedicine	3.97	78	3.12	38	3.85	72	8.85^*^
I would like to continue conducting outpatient telemedicine visits in the future	3.91	70	3.23	46	3.96	70	4.02^*^

^a^ Average Likert score (1–5).

^b^ % Strongly agree or agree.

^*^ *p* < 0.05.

They also differ on safety perceptions when using telemedicine with inpatient (*F* = 11.32, *p* < 0.05) and outpatient (*F* = 8.85, *p* < 0.05) visits and perceived quality of inpatient care (*F* = 3.55, *p* < 0.05) and outpatient care (*F* = 4.02, *p* < 0.05).

Similar patterns existed for outpatient telemedicine use, with APPs reporting the best satisfaction scores, and nurses reporting the lowest, though physician satisfaction scores were higher for outpatient telemedicine at 60% than for inpatient telemedicine (Table 4). More than 50% of APPs (*n* = 65) and physicians (*n* = 36) indicated greater flexibility in how work is conducted as a major benefit of telemedicine (Table 2). However, respondents indicated frustration with pre-scheduling and scheduling activities, and reduced personal or emotional connection with patients.

Nearly 20% (*n* = 18) of nurses reported difficulty learning new apps and processes and difficulty with Internet or network connections as major frustrations (Table 5). Seventy percent of APPs and physicians (*n* = 38) wanted to continue conducting outpatient telemedicine visits in the future, whereas only 46% of nurses reported the same.

**Table 5.** Percentage of Advanced Practice Providers’, Nurses’, and Physicians' Perceived Positive Changes Due to Telemedicine Use

	APP	Nurse	Physician	Total
Number of observations	118	49	70	237
% Reducing exposure time to COVID-positive or presumed positive patients^a^	69	53	63	64
% Reducing PPE use^a^	59	49	54	56
% Greater flexibility in how I conduct my work^a^	55	37	51	50
% Patient satisfaction with telemedicine^a^	34	12	41	32
% Easier to coordinate with family members or caregivers^a^	29	29	26	28
% More efficient^a^	31	10	24	24
% Scheduling^a^	20	12	7	15
% Patient adaptability to technology^a^	16	6	13	13
% Visits are technologically easy to conduct^a^	15	4	11	12
% Integration across apps and programs^a^	8	4	1	5
% Using tablets^a^	1	4	3	2
Average count of telemedicine positive changes	3.5	2.3	3.1	3.1

^a^ Percent of health care workers who marked yes.

PPE, personal protective equipment.

I have been pleasantly surprised with how much I enjoyed seeing patients via telemedicine. [APP]

Telehealth has been a very positive addition to my practice, in addition to working from home more on administrative days—and still being productive! [Physician]

### Safety

One of the aims of rapid scaling of telemedicine was to prevent unnecessary exposure to COVID-19 and conserve PPE. Physicians (*n* = 70, 54%), APPs (*n* = 24, 59%), and nurses (*n* = 38, 49%) cited reduction of PPE use as a benefit of telemedicine (Table 5). A majority of respondents (*n* = 152, 64%) also indicated that telemedicine reduced their exposure time to COVID-positive or presumed positive patients. Likewise, when asked whether they felt safer having the option to use inpatient telemedicine to treat patients during COVID-19, 75% (*n* = 21) of APPs and 64% (*n* = 11) of physicians agreed. It is noteworthy that only 29% (*n* = 6) of nurses reported feeling safer.

Analysis of the qualitative responses suggested that nurses were often asked to go into the room to help the patient use the telemedicine technology, often sparing the doctor or APP from entering. This created a sense that reducing the COVID-19 exposure for the physician by using inpatient telemedicine was often done at the expense of the nurse:

Providers don't consider the amount of work behind setting an inpatient up to use telemedicine, and often times ask the RN to go into the room to connect the device so the physician does not have to enter the room… The nursing staff feels as if they are “sacrificed” to go into the COVID positive rooms in place of a physician.—[Nurse]

We have had ongoing issues with other disciplines inappropriately asking nurses to use PPE to help patients to use devices so they don't have to enter the room… [Nurse]

Doctors should not rely on nurses to go into rooms for them when telemedicine is not working. [Nurse]

A physician seemingly confirmed this dynamic by suggesting that other staff could manage arranging visits with the patients' family so their rounds would not be delayed:

We should have a standard system that allows me to interact with the family members when I see an inpatient… It could be facilitated by social workers or nurses so my patient rounds are not delayed.—[Physician]

### Patient care and satisfaction

The findings were mixed on the perceived relationship between patient care, patient satisfaction, and telemedicine. Clinicians were less satisfied with the quality of care that patients were receiving for inpatient telemedicine versus outpatient telemedicine (Table 2). Within outpatient telemedicine, clinicians were more satisfied with the quality of care that existing patients were receiving relative to new patients. More than one-third of physicians expressed concern with difficulty in diagnosis via telemedicine (37%, Table 6).

**Table 6.** Percentage of Advanced Practice Providers’, Nurses’, and Physicians' Perceived Telemedicine Frustrations

	APP	Nurse	Physician	Total
Number of observations	118	49	70	237
% Patient connectivity issues (poor Internet or network connections)^a^	58	45	63	57
% Patient inability to use the technology as intended^a^	50	47	61	53
% Patient frustration with Telemedicine^a^	32	45	33	35
% Continued difficulty with telemedicine apps and processes^a^	28	37	39	33
% Difficulty coordinating among multiple care team members^a^	19	35	33	26
% Difficulty in diagnosing via telemedicine (difficult to see, feel, check vitals)^a^	24	20	37	27
% Pre-scheduling activities^a^	27	24	27	27
% Reduced personal/emotional connection with patients^a^	26	29	27	27
% Scheduling^a^	27	18	30	26
% Difficulty learning new apps and processes for telemedicine at the beginning^a^	19	27	20	21
% Personal connectivity issues (poor Internet or network connections)^a^	17	27	17	19
% Difficulty accessing a secure connection or logging in remotely^a^	8	12	6	8
Problems with integration across apps^a^	4	6	11	7
Average count of telemedicine frustrations	3.5	3.6	4.4	3.8

^a^ Percent of health care workers who marked yes.

More than 30% of nurses (*n* = 17) and physicians (*n* = 32) reported difficulty coordinating among multiple care team members when using telemedicine (Table 4). However, nearly 30% (*n* = 66) of team members also reported greater ease in coordinating with family members and caregivers. In terms of patient satisfaction, nurses were less likely to indicate that they perceived patient satisfaction with telemedicine was a positive outcome of telemedicine, and more likely to indicate patient frustration as a challenge with telemedicine (Tables 4 and 6). In the qualitative responses, respondents indicated that they believed telemedicine was a benefit to patient care, particularly as it allowed continuity to health care access during the early phases of COVID-19:

Telemedicine has allowed for patients to be seen during uncertain times/when social distancing is recommended. [APP]

Although difficult to adapt to for my patient population, the evolution of telemedicine was quick and great for patient care during the height of crisis. [APP]

Telemedicine. It's been absolutely game-changing for a lot of our patients. [Physician]

However, some believed that telemedicine was inappropriately used in place of an in-person visit:

Quit using them as a way to NOT come see and assess the patients. Too many physicians rely on this and it's unacceptable. [Nurse]

### Patient factors

Patient-level factors were also indicated as a source of frustration with telemedicine. More than half of clinicians (53%) indicated frustration with patients' inability to use the technology as intended (Table 6). In addition, patient connectivity issues such as poor Internet or network connections were cited as a major source of frustration for clinicians. (*n* = 135, 57%) However, some did cite patient adaptability to technology as a benefit of telemedicine (*n* = 31, 13%).

## Discussion

The COVID-19 pandemic pressured health systems to increase their usage of telemedicine to continue caring for patients while reducing the spread of the virus and balancing PPE. Although the pandemic will eventually end, the existence of inpatient telemedicine holds promise for the future in terms of expanded access to care and increased flexibility for providers. Outpatient telemedicine has the potential to drastically expand access to health services and increase flexibility and autonomy in the work environment for providers.

Inpatient telemedicine holds promise for equipping health systems to be more resilient in the face of current and future infectious diseases. The rapid changes during the pandemic resulted in a relaxation of payment and regulatory policies governing the adoption of and incentives for telemedicine.^26^ Many expect these policies to continue beyond the pandemic, paving the way for both inpatient and outpatient telemedicine to become an enduring part of the way we deliver care to patients.

Although recent studies have highlighted the benefits of telemedicine, including expanded access and flexibility,^27^ our study also highlights the potential for telemedicine to be a mechanism of improved work satisfaction for some users. Specifically, respondents indicated increased autonomy and flexibility in their schedules as major benefits. Health care has historically offered its workers rigid and inflexible shift-based schedules, which allows little flexibility to balance the demands of life outside of work. Telemedicine may be a tool for adding more freedom for the provider to work around their many competing demands at work and at home, as indicated in the qualitative results.

As this method of care delivery continues to expand, it is important to understand both the benefits and what additional burdens, if any, are placed on the clinical users. However, despite its growing adoption, much of the work on telemedicine has focused on patient experiences with telemedicine, with some studies examining physician, nurse, or APP experiences in a siloed manner.^28,29^ However, our study highlights the importance of considering the entire team together when evaluating the adoption and implementation of telemedicine. Specifically, our results indicate that greater satisfaction for some members of the care team may come at the expense of others, namely nurses.

A siloed approach to understanding user satisfaction with telemedicine only presents a partial picture of the range of experiences that may exist with the implementation of such tools. This has implications for the adoption of other technologies within health care as well. The assumption may be that the main end user is the most critical stakeholder when evaluating satisfaction with the new technology, without recognizing the differing impacts it may have throughout the team.

For example, one of the main stated goals of implementing inpatient and outpatient telemedicine was to reduce exposure to COVID-positive patients and to conserve PPE at a time where PPE supply was uncertain. In this regard, the results of our study suggest that the implementation of telemedicine did accomplish some of these goals. The majority of respondents believed that telemedicine reduced the use of PPE and reduced their exposure to COVID-positive or presumed-positive patients.

A majority of physicians and APPs felt safer being able to use telemedicine, whereas less than a third of nurses felt the same. The qualitative results indicated that nurses were often sent into the room unnecessarily, solely for the purposes of setting up the patient for inpatient telemedicine. Nurses felt that their safety was compromised to protect the safety of other team members.

Many of our respondents noted that adapting to telemedicine was both challenging and positive. Organizations should not be dissuaded by early friction in transitioning to new modalities, but rather should adopt mechanisms for collecting rapid feedback during the implementation phases to make adjustments and reduce frustration where possible. In addition, our results indicate that the health care organization does not bear all of the responsibility for frustration with telemedicine.

For example, the top 3 most frequent frustrations with telemedicine were outside the scope of the organization's control. The top frustration was related to issues with the patient's connectivity or poor Internet or network connections. This health system serves a rural patient population and has notable poor network infrastructure throughout its state. Even the most successful implementation and perfectly designed telemedicine interface cannot surmount this issue. To develop better capacity throughout the referral region, state and industry partnerships are needed to expand the infrastructure to support the growth of telemedicine. Similarly, the technology literacy of the patient population can also be a major frustration for clinicians using telemedicine.

Although these study findings highlight the importance of evaluating telemedicine experiences across the entire health care team, there are some limitations. First, this study was conducted within a single hospital and thus the findings may not be generalizable to other settings. In addition, there is the possibility of a nonresponse bias, meaning those who responded to the survey differ from those who did not. Lastly, this study was conducted during a rapid implementation of both inpatient and outpatient telemedicine programs during a pandemic.

Therefore, caution must be exercised in assuming that the frustrations mentioned herein are persistent or unavoidable under a normal implementation schedule and without the additional stressors of health care work during a pandemic. In fact, the many positive responses to telemedicine despite the stresses of the pandemic may hold promise for the future of telemedicine.

## Conclusion

In conclusion, these results suggest that telemedicine holds promise for providing care beyond the pandemic, and it may be a mechanism for increasing autonomy, flexibility, and satisfaction with work. To the degree that it can be improved now and beyond the pandemic, it can increase the resilience of health systems to deal with other infectious diseases, as well as increase patient access to health services. However, the implementation of any such innovation needs to carefully and thoughtfully consider the experiences of the entire team that will be affected by the new technologies, rather than a siloed approach to determining satisfaction with the changes.

## Abbreviations Used

%: percent within the job role group and total sample
ANOVA: analysis of variance
APP: advanced practice provider
CI: confidence interval
ICU: Intensive Care Unit
M: mean
PPE: personal protective equipment
SD: standard deviation
